# Evaluating the Diagnostic Potential of Chorismate Mutase Poly-Clonal Peptide Antibody for the *Acanthamoeba* Keratitis in an Animal Model

**DOI:** 10.3390/pathogens12040526

**Published:** 2023-03-28

**Authors:** Min-Jeong Kim, Hye-Jeong Jo, Hae-Jin Sohn, Ho-Joon Shin, Fu-Shi Quan, Hyun-Hee Kong, Eun-Kyung Moon

**Affiliations:** 1 Department of Biomedical Science, Graduate School, Kyung Hee University, Seoul 02447, Republic of Korea; 2 Department of Microbiology, Ajou University School of Medicine, Suwon 16499, Republic of Korea; 3 Department of Medical Zoology, School of Medicine, Kyung Hee University, Seoul 02447, Republic of Korea; 4 Medical Research Center for Bioreaction to Reactive Oxygen Species and Biomedical Science Institute, Core Research Institute (CRI), Kyung Hee University, Seoul 02447, Republic of Korea; 5 Department of Parasitology, Dong-A University College of Medicine, Busan 49201, Republic of Korea

**Keywords:** *Acanthamoeba* keratitis (AK), AK animal model, chorismate mutase (CM), polyclonal peptide antibody

## Abstract

*Acanthamoeba* spp. is the causative agent of *Acanthamoeba* keratitis (AK), a vision-threatening parasitic disease whose primary risk factor has been attributed to poor contact lens hygiene. Unfortunately, differential diagnosis of AK is challenging as the clinical manifestations for AK are similar to those of bacterial, fungal, or even viral keratitis. Since delayed AK diagnosis can incur permanent vision impairment, a rapid and sensitive diagnostic method is urgently needed. Here, the diagnostic potential of polyclonal antibodies targeting the chorismate mutase (CM) of *Acanthamoeba* spp. was evaluated in AK animal models. CM antibody specificity against *Acanthamoeba* trophozoites and cysts was confirmed by immunocytochemistry after co-culturing *Acanthamoeba* with *Fusarium solani*, *Pseudomonas aeruginosa*, and *Staphylococcus aureus*, and human corneal epithelial (HCE) cells. Enzyme-linked immunosorbent assay (ELISA) was performed using CM-specific immune sera raised in rabbits, which demonstrated that the antibodies specifically interacted with the *Acanthamoeba* trophozoites and cysts in a dose-dependent manner. To evaluate the diagnostic potential of the CM antibody, AK animal models were established by incubating contact lenses with an inoculum containing *A. castellanii* trophozoites and subsequently overlaying these lenses onto the corneas of BALB/c mice for 7 and 21 days. The CM antibody specifically detected *Acanthamoeba* antigens in the murine lacrimal and eyeball tissue lysates at both time points. Our findings underscore the importance of antibody-based AK diagnosis, which could enable early and differential AK diagnosis in clinical settings.

## 1. Introduction

*Acanthamoeba* spp. is a free-living amoeba found in the environment which can cause opportunistic infections in humans [1,2,3]. One such disease associated with *Acanthamoeba* infection is *Acanthamoeba* keratitis (AK), a rare but vision-threatening disease associated with contact lens wear [4,5,6,7]. Since its first report in 1973, the prevalence of AK has been steadily increasing throughout the globe [8,9,10]. Despite this ongoing trend, accurate AK diagnosis has remained quite challenging as the clinical symptoms for AK are similar to those of other corneal infections, which include ophthalmalgia and corneal ring infiltration [11,12]. Diagnosis is further hindered by the diverse microbial flora that cohabits the identical niche along with *Acanthamoeba* spp., especially *Pseudomonas* spp., or *Fusarium* spp. as these are opportunistic pathogens associated with contact lens usage [13,14]. Given this circumstance, a method that rapidly and selectively differentiates AK from keratitis of other microbial origins is of significant importance as prolonged *Acanthamoeba* spp. exposure to the corneas permeates their penetration into the stromal layer, which consequently leads to corneal ulcers and permanent vision impairment [15]. Penetration of this pathogen into the stromal layer causes further treatment difficulties and for this reason, a rapid and accurate diagnostic method is highly desired. Developing such a technique would not only enhance AK diagnosis but also ensure adequate treatment is received on time to prevent further exacerbation of the disease.

Currently, the conventional diagnostic method of AK includes clinical specimen cultivation, confocal microscopy, polymerase chain reaction, and stains [16,17,18,19,20,21,22]. Indubitably, these highly accurate diagnostic methods are considered to be the gold standard for AK diagnosis, but the major downsides of these techniques are the reliance on invasive sample acquisition technique. For example, as a consequence of corneal scraping for human sample procurement, the patients are likely to experience immense pain during the process. Furthermore, these diagnostic tests may provide false negative results if the sample quantity is insufficient. Microscopic analysis and staining are other well-established techniques for AK diagnosis with minor drawbacks. While in vivo confocal microscopy of *Acanthamoeba* cysts have clearly delineated the presence of double-walled structures, trophozoites lack this structure and are difficult to distinguish from leukocytes. Additionally, while staining with Giemsa, calcofluor white and acridine orange usually give very good results, cysts are prone to exhibiting autofluorescence and some strains of fungi can be stained by them, which renders diagnosis somewhat difficult. Moreover, *Acanthamoeba* cultures vary in sensitivity depending on the culture technique and generally do not provide immediate results. For these reasons, it is necessary to develop a non-invasive diagnostic tool that is painless, easy to perform, and can rapidly diagnose AK. One potential approach to non-invasive AK diagnosis is the use of *Acanthamoeba*-specific antibodies. To date, numerous immunological assays investigating the *Acanthamoeba* antigen-antibody interactions have been conducted. Previously, in a survey conducted in New Zealand, a high prevalence of *Acanthamoeba*-specific antibody titers was reported in all of the asymptomatic individuals tested in the study, thus suggesting that humans are commonly exposed to *Acanthamoeba* [23]. Consistent with this finding, enzyme-linked immunosorbent assay (ELISA) performed in the Saudi Arabian population revealed the presence of *Acanthamoeba*-specific secretory IgA (sIgA) in the mucosal samples, and its prevalence rate in the tested individuals exceeded 80% [24]. Interestingly, when a group of healthy individuals and AK patients were tested for the presence of anti-*Acanthamoeba* antibodies, AK patients showed significantly lower levels of slgA than healthy individuals [25]. Therefore, diagnosing AK based on the presence of *Acanthamoeba*-specific antibodies in sera would be misleading. On the contrary, designing an antibody that specifically interacts with *Acanthamoeba* spp. antigens from clinical specimens would prove to be useful.

In one study, four antibodies with high specificity to *Acanthamoeba* were isolated through extensive screening of more than 1700 clones from the bacteriophage display library [26]. However, neither cross-reactivity with other causative agents of keratitis nor the minimum concentration required to detect *Acanthamoeba* were evaluated in the aforementioned study. Recently, eight monoclonal antibodies against *Acanthamoeba* (AMEC1-3, MTAC1-3, MTC4, and MTAC5) were produced [27]. Among them, six antibodies (AMEC1-3 and MTAC1-3) reacted only to cysts while MTC4 reacted to both trophozoites and cysts. MTAC5 reacted weakly with trophozoites in flow cytometric analysis. Yet, there is a relative paucity of in vivo studies confirming the *Acanthamoeba*-specificity of antibodies raised for AK diagnosis.

To address this limitation, we recently reported the diagnostic potential of *Acanthamoeba*-specific adenylyl cyclase-associated protein (ACAP) and periplasmic binding protein (PBP) antibodies using the tears and eyeball lysates acquired from the AK mouse model [28]. While this study demonstrated that non-invasive diagnosis of AK via antibody-based methods is feasible, both ACAP and PBP antibodies weakly interacted with the murine samples. To improve the diagnostic potential of antibody-based AK diagnostic methods, we tested the specificity and sensitivity of the chorismate mutase (CM) antibody which elicited substantially higher antibody responses [29]. Our findings outline several improvements to the non-invasive AK diagnosis methods which could aid their further development.

## 2. Materials and Methods

### 2.1. Preparation of Cell Cultivation

*Acanthamoeba castellanii* (ATCC 30868) and human corneal epithelial (HCE) cells (ATCC PCS-700-010) were obtained from the American Type Culture Collection (Manassas, VA, USA). *A. castellanii* trophozoites were aseptically cultured with Peptone Yeast Glucose (PYG) media at 25 °C. Trophozoites of *Acanthamoeba* were subcultured at 70% or greater cell confluence. HCE cells were cultured in a 100 mm petri dish until confluent at 37 °C with CO_2_ in endothelial cell growth medium kits (KGM BulletKit, Lonza, Portsmouth, NH, USA). *Fusarium solani* (NCCP 32678), *Pseudomonas aeruginosa* (NCCP 16091), and *Staphylococcus aureus* (NCCP 15920) were obtained from the Korea Centers for Disease Control and Prevention. *F. solani* was cultured in Sabouraud Dextrose (SD) media at 25 °C for 2 days, while *P. aeruginosa* and *S. aureus* were cultured in Brain Heart Infusion (BHI) media at 37 °C for 1 day.

### 2.2. Encystation Assays

*A. castellanii* trophozoites (5 × 10^5^ cells) were collected and washed with phosphate-buffered saline (PBS). Encystation of *A. castellanii* trophozoites was induced using encystment media (95 mM NaCl, 5 mM KCl, 8 mM MgSO_4_, 0.4 mM CaCl_2_, 1 mM NaHCO_3_, and 20 mM Tris-HCl, pH 9.0) at 25 °C for 2 days as previously described [30]. The morphological transformation of trophozoites to mature cysts was verified under the microscope.

### 2.3. Immunocytochemistry

After placing sterile coverslips in a 6-well plate, HCE cells (3 × 10^5^ cells/well) were incubated overnight in KGM Bulletkit medium at 37 °C with CO_2_. The next day, HCE cells were co-cultured with *A. castellanii* trophozoites and cysts (5 × 10^5^ cells/well) at 37 °C for 4 h. *F. solani*, *P. aeruginosa*, and *S. aureus* were cultured until the early exponential phase (OD_600nm_ = 0.8) and incubated with HCE cells and *A. castellanii* for 1 h. After discarding the culture media, cells were washed three times with ice-cold autoclaved PBS and then fixed with cold methanol for 5 min at RT. After repeated washing with PBS for 5 min, cells were incubated in a blocking buffer solution containing 1% BSA and 22.5 mg/mL glycine in PBS with 0.05% Tween 20 (PBST) for 30 min at RT. Afterward, cells were incubated overnight at 4 °C with CM antibody (1:200 dilution in blocking buffer). The next day, cells were washed with PBS and probed with CruzFluorTM 555 (CFL-555)-conjugated anti-rabbit IgG (1:400 dilution in blocking buffer) (Santa Cruz Biotechnology, Dallas, TX, USA) for 2 h at RT. After washing with PBS, cells were stained with VECTASHIELD mounting medium containing 4′,6-diamidino-2-phenylindole (DAPI) (Vector Laboratories, Newark, CA, USA) and observed under a fluorescence microscope (Leica, Wetzlar, Germany).

### 2.4. Enzyme-Linked Immunosorbent Assay (ELISA)

ELISA was performed to evaluate *Acanthamoeba* CM-specific antibody response and minimum detectable concentration of the *Acanthamoeba* antigens. Cell lysates of *A. castellanii* trophozoites and cysts were prepared. Cell lysates were serially diluted 10-fold to assess optimal detection concentrations (200 to 0.0002 μg/mL). For ELISA, 96 well plates were coated with cell lysates diluted in coating buffer (0.1 M sodium carbonate, pH 9.5) and incubated overnight at 4 °C. Between each incubation step, plates were washed three times with PBST. After blocking the wells with 0.2% gelatin in PBST at 37 °C for 2 h, CM antibody (1:200 dilution in PBS) was inoculated into each well and the plate was incubated at 37 °C for 1 h. Horseradish peroxidase (HRP)-conjugated anti-rabbit IgG, IgA (Sigma-Aldrich, St. Louis, MO, USA), and IgM (Southern Biotech, Birmingham, AL, USA) (1:1000 dilution in PBS) were added to each well and incubated at 37 °C for 1 h. O-phenylenediamine (OPD) substrate was dissolved in citrate-phosphate buffer (pH 5.0) containing 30% H_2_O_2_ and added to each well. The plate was read at 450 nm using an EZ Read 400 microplate reader (Biochrom Ltd., Cambridge, UK). The sera from unimmunized mice were used as the negative control.

### 2.5. Animals and Ethics Requirements

For this experiment, 19 eight-week-old female, BALB/c mice (Orient Bio, Seongnam, Republic of Korea) were used. All experiments involving animals were conducted in adherence to the Animal Research: Reporting of In Vivo Experiments (ARRIVE) guidelines. All animals were maintained in specific-pathogen-free (SPF) conditions approved by the Association for Assessment and Accreditation of Laboratory Animal Care (AAALAC). All experimental procedures involving animals were approved by the Institutional Animal Care and Use Committee (IACUC) of Ajou University Medical Center (AUMC-IACUC-2021-0006). Animals were housed in approved facilities with 12 h day and night cycles with easy access to food and water *ad libitum*. To minimize animal suffering, the body weights of mice were measured daily and weight loss exceeding 20% of the initial values was considered to be the humane intervention point. Mice reaching this intervention point were humanely euthanized by CO_2_.

### 2.6. Establishment of an Animal Model for Acanthamoeba Keratitis

*A. castellanii* trophozoites were used to induce keratitis in BALB/c mice as described previously [31]. Briefly, *A. castellanii* trophozoites were centrifuged (at 1500 rpm for 3 min) to collect a concentration of 1 × 10^5^ cells. A commercial soft contact lens (Proclear 1-Day Contact Lenses, CooperVision, NY, USA) was cut into 2 mm circles using a hole punch. *A. castellanii* trophozoite pellet was resuspended in 20 μL of sterile PBS, which was gently overlaid on the surface of the contact lens and incubated at 25 °C for 1 h. Mice were anesthetized by intraperitoneal injection of ketamine and xylazine solution (5 mg/mL ketamine and 1 mg/mL xylazine combined; 0.3 mL/20 g), and several incisions were made on the corneal surface using a scalpel and 25-gauge syringe needle. A contact lens inoculated with *A. castellanii* trophozoites was placed on the cornea where the incisions were made, and the eyelids were sutured with 6-0 nylon sutures (Woorimedical, Namyangju, Republic of Korea). The eyes of the mice were continuously monitored to assess keratitis development. To collect tear samples and eyeball tissues, mice were appropriately anesthetized on the 7 (*n* = 7) and 21 days (*n* = 7) post-infection (dpi), and the inoculated lens was removed by opening the eyelid sutures. A total of 100 μL was collected by repeatedly washing the corneal surface with 10 μL of sterile PBS. The whole eyeball tissues were homogenized in 500 μL of sterile PBS. Naïve mice were also run in parallel (*n* = 5). All samples were stored at −80 °C until use.

### 2.7. Detection of Antigen in AK Animal Model Using CM Antibody

ELISA was used to detect *Acanthamoeba* antigens in tear samples and eyeball lysates acquired from the AK mice model using CM antibody. Briefly, tear samples (5 μg/mL) and whole eyeball tissue lysates (5 μg/mL) of naïve mice and AK animal models were coated overnight at 4 °C with carbonate coating buffer in 96-well plates. The plate was washed with PBST and blocked with 0.2% gelatin at 37 °C for 2 h. Diluted CM antibodies (1:250 dilution in PBST for tear samples and 1:1250 dilution in PBST for eyeball tissue lysates) were inoculated into respective wells, and plates were incubated at 37 °C for 1 h. HRP-conjugated anti-rabbit IgG, IgA, and IgM antibodies were diluted 1:2000 in PBST and incubated at 37 °C for 1 h. Color development was conducted by inoculating 100 μL of substrate buffer containing OPD and H_2_O_2_ into each well and OD_450_ values were measured using a microplate reader.

### 2.8. Statistical Analysis

All statistical analyses were performed using GraphPad Prism version 8.0 (San Diego, CA, USA). Data were presented as the mean ± SD, and statistical significance between the groups was determined by Student’s *t*-test and denoted using an asterisk. *p* < 0.05 was considered statistically significant (* *p* < 0.05, ** *p* < 0.01, *** *p* < 0.001, and **** *p* < 0.0001).

## 3. Results

### 3.1. Confirmation of CM Antibody Specificity against Acanthamoeba Trophozoites and Cysts

**Figure 1:**
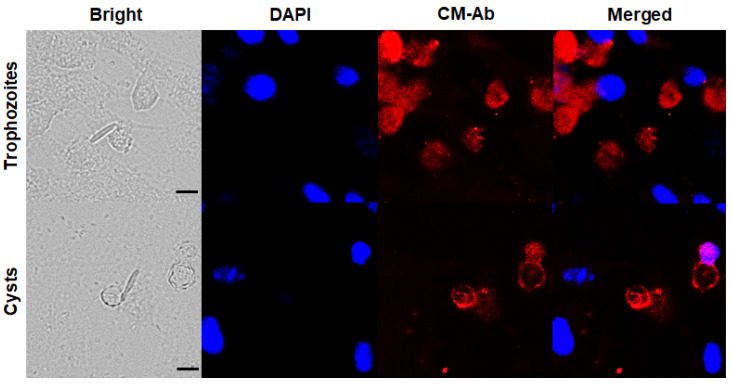
In vitro confirmation of chorismate mutase (CM)-Ab specificity via immunocytochemistry. *A. castellanii* trophozoites and cysts were co-cultured with HCE cells, *F. solani*, *P. aeruginosa*, and *S. aureus* and observed under a fluorescent microscope. Cells were incubated with CM antibody and stained with DAPI mounting medium. Bright-field, DAPI (blue), CM antibody (red), and merged images were acquired at 400 × magnification. The black scale bar denotes 10 μm.

To confirm CM-specific antibody response against trophozoites and cysts of *A. castellanii*, immunocytochemistry was performed. *A. castellanii* trophozoites specifically interacted with the CFL-555 labeled CM antibody and they were visualized as red under a fluorescence microscope (Figure 1, upper panels). *A. castellanii* cysts were also clearly visualized in red (Figure 1, lower panels), and it was confirmed that the CM antibody strongly interacted with *Acanthamoeba* cysts. The nuclei of HCE cells were stained with DAPI (blue). *F. solani*, *P. aeruginosa*, and *S. aureus* responded weakly to DAPI, but not to the CM antibody. These results show that the CM antibody of *A. castellanii* can specifically detect both *A. castellanii* trophozoites and cysts.

### 3.2. Determining the Minimum Detectable Concentration of Acanthamoeba Antigens by CM Antibody

**Figure 2:**
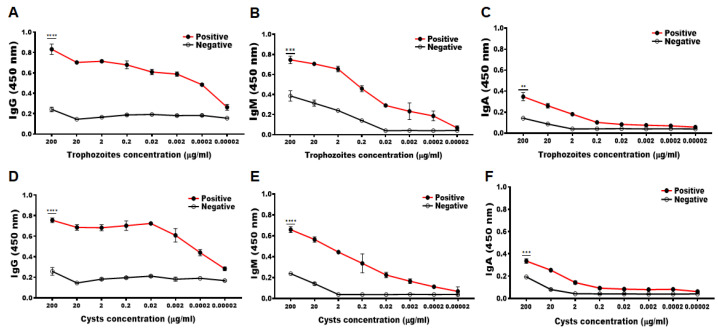
Confirmation of specific antibody responses against *Acanthamoeba* trophozoites and cysts. The CM-specific IgG, IgM, and IgA antibody titers were determined using serially diluted *A. castellanii* trophozoites (**A**–**C**) and cysts (**D**–**F**) lysates. Asterisks denote statistically significant differences between positive (red lined ●) and negative (black lined ○) serum (** *p* < 0.01, *** *p* < 0.001, and **** *p* < 0.0001).

To determine the minimum detectable concentration of antigens from *A. castellanii* trophozoite and cysts, ELISA was performed using immunized rabbit serum. The serum immunized with the CM peptide antigen of *A. castellanii* was found to react specifically with the cell lysates of *A. castellanii* trophozoites and cysts (Figure 2). Compared to the negative control group, a marked increase in serum CM-specific IgG, IgM, and IgA was observed against *Acanthamoeba* trophozoites up to dilutions of 0.0002, 0.2, and 2 µg/mL (Figure 2A–C), and *Acanthamoeba* cysts up to dilutions of 0.0002, 0.2, and 20 µg/mL each, respectively (Figure 2D–F). Based on these findings, CM antibody was determined to be highly specific to *A. castellanii* trophozoites and cysts, which can detect the *Acanthamoeba* even when it is present at low concentrations.

### 3.3. Construction of the AK Animal Model

**Figure 3:**
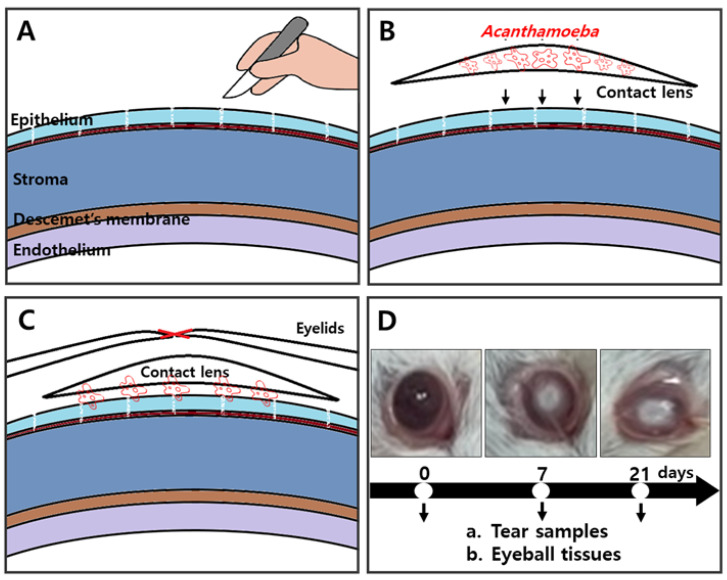
Experimental design for AK animal models. After scratching the corneas of BALB/c mice (**A**), contact lenses incubated with *A. castellanii* trophozoite (1 × 10^5^ cells) inoculum were allowed to adhere to the corneal surface (**B**). Suturing of eyelids to ensure prolonged physical contact and prevent contact lenses from falling off (**C**). Experimental scheduling for tear samples and eyeball tissue acquisition from AK-induced mice on day 7 (*n* = 7) and day 21 (*n* = 7) (**D**). Naïve mice were also run in parallel (*n* = 5).

To evaluate the *Acanthamoeba* antigen detection capabilities of CM antibodies in vivo, AK mouse models were established. A schematic diagram portraying the procedures for the establishment of an AK mouse model was provided (Figure 3). After scraping the cornea of the mice, a contact lens attached with *A. castellanii* trophozoites was inoculated and the eyelids were sutured (Figure 3A–C). Sutures were removed on the 7 and 21 days after infection. Compared to naïve mice, inflammation and ring ulcers typical of AK symptoms were developed on both 7 and 21 days. (Figure 3D). At 7 and 21 dpi, lacrimal samples and eyeball tissues were harvested for ex vivo analyses.

### 3.4. Detection of Acanthamoeba Antigens in the AK Animal Model

**Figure 4:**
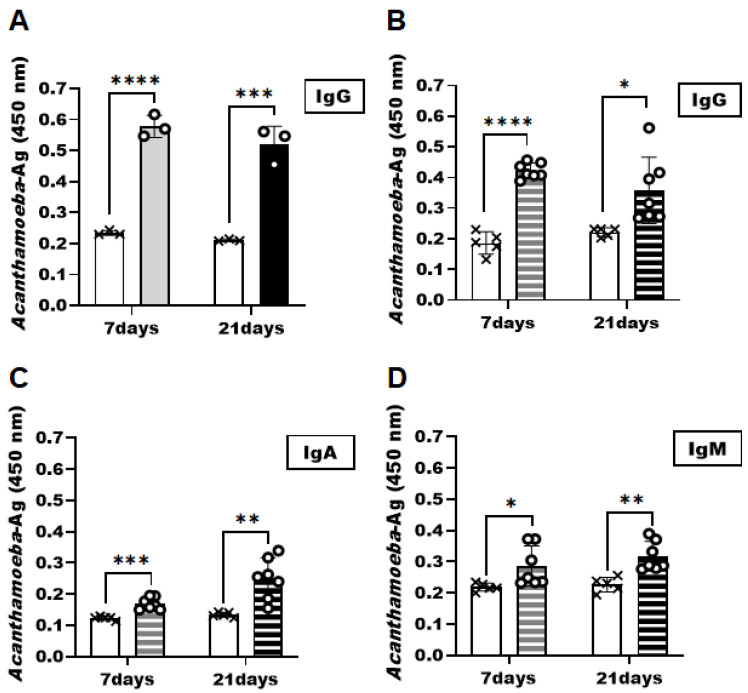
Detection of *Acanthamoeba* antigens from the tear samples and eyeball tissue lysates by CM antibody. At 7 and 21 days after inoculation with *A. castellanii* trophozoites, the eyelids were unsutured and tear samples and eyeball tissue lysates were collected. The detection of *Acanthamoeba* antigens was investigated by the CM antibody of *A. castellanii*. *Acanthamoeba* antigens in tear samples were detected by anti-*Acanthamoeba* IgG (**A**). *Acanthamoeba* antigens detection in the eyeball tissue lysates was determined using anti-*Acanthamoeba* specific IgG, IgA, and IgM antibody (**B**–**D**). Negative (×); naïve-mouse tear samples and eyeball tissue lysates, positive (○); AK-mouse tear samples and eyeball tissue lysates. Data are expressed as mean ± SD and asterisks denote statistically significant differences between positive and negative groups (* *p* < 0.05, ** *p* < 0.01, *** *p* < 0.001, and **** *p* < 0.0001).

To demonstrate that the *Acanthamoeba* specificity of the CM antibody observed in vitro is also applicable in vivo, ELISA was performed using the samples acquired from the AK mice model at 7 and 21 dpi (Figure 4). CM antibody successfully interacted with the *Acanthamoeba* antigens in tear samples acquired at both infection time points (Figure 4A). Compared to the control group, significantly greater *Acanthamoeba*-specific antibody responses were elicited in the AK mice model. However, significant differences in antigen-specific antibody responses were not observed in the AK mice samples between the two sampling time points. Consistent with the results of the tear samples, *Acanthamoeba*-specific antibody responses were also detected in the eyeball tissues of the AK animal model (Figure 4B–D). The eyeball tissue lysates of the AK model collected on 7 and 21 dpi were substantially higher than those of their naïve counterparts. *Acanthamoeba*-specific IgG levels detected using the CM antibody in eyeball lysates were comparable to those observed in tear samples (Figure 4B). However, though significantly greater than the controls, IgA (Figure 4C) and IgM (Figure 4D) responses were elicited to a weaker extent than the IgG in the murine AK samples.

## 4. Discussion

Developing a non-invasive diagnostic method for AK that provides accurate results in a relatively short time frame has been challenging. Here, in a follow-up study to our previous works [29] we investigated the feasibility of CM antibody as an antibody-based AK diagnostic tool in animal models. Our findings revealed that the CM antibody strongly and selectively interacted with both *A. castellanii* trophozoites and cysts with complete disregard for other microbial pathogens present in the vicinity that are capable of causing keratitis. The antibody was capable of detecting *Acanthamoeba* at a concentration as low as 0.0002 μg/mL and successfully detected *Acanthamoeba* antigens in murine AK samples.

Previously, we reported that *Acanthamoeba* antigens in tears and eyeball lysates of the AK mouse model were detected by adenylyl cyclase-associated protein (ACAP) and periplasmic binding protein (PBP) antibodies [28]. Interestingly, *Acanthamoeba* antigens in the AK mice samples were detected to a greater extent by the CM antibody in comparison to the ACAP and PBP reported in our previous study. Such a phenomenon was observed in both lacrimal and eyeball lysate samples. Although the underlying factors accounting for this discrepancy remain unknown, we speculate that CM possesses relatively greater antibody avidity than ACAP and PBP. In support of this notion, the protein expression data acquired via western blot in our previous studies revealed that CM antibody was capable of detecting *Acanthamoeba* antigens in both cell lysates and their conditioned media [29]. On the contrary, ACAP and PBP were only detectable from the cell lysate samples [32,33]. Because the ocular acquired from mice contains both *Acanthamoeba*, as well as their secretory products, an antibody with greater avidity for *Acanthamoeba* antigens may have contributed to substantial differences in ELISA OD values for these antibodies.

In the present study, extremely minuscule quantities of tears were observed in mice, which were further diluted with PBS during the sample acquisition process. Despite this, noticeable differences in antigen-specificity were observed when compared to the controls. Based on this finding, CM antibody-based *Acanthamoeba* antigen detection using lacrimal samples could be sufficient for accurate AK diagnosis. Furthermore, because AK patients are prone to produce excessive amounts of tears [34,35], tear dilution would not be needed as was the case in murine models. One major limitation of this study is the lack of data assessing the potential of CM, the antibody against chronic AK. However, since *Acanthamoeba* migrates into deeper eye layers as time progresses [5,36], we anticipate that diagnosis with antibody-based methods using tear samples could be difficult in the chronic stage. Here, 21 dpi was the endpoint of the study and ocular samples from mice where AK was induced for months were not tested, as we were primarily focused on early-stage AK diagnosis. Further studies evaluating these aspects would prove to be useful for improving antibody-based AK diagnostic methods.

In summary, given the high *Acanthamoeba* antigen-specificity demonstrated using murine samples, the polyclonal CM antibody described here could be further developed as a candidate antibody for AK diagnosis. Since the CM antibody specifically detected the antigen in tear samples and eyeball tissue lysates of the AK-induced mice model, this is expected to have a significant impact on the development of non-invasive and rapid immunological diagnostic methods.

## Data Availability

Data supporting the findings of this study are contained within the article.
